# Zinc Deficiency Exacerbates Lead-Induced Interleukin-2 Suppression by Regulating CREM Expression

**DOI:** 10.3390/ijms26010254

**Published:** 2024-12-30

**Authors:** Hannah E. Trojan, Lothar Rink, Jana Jakobs

**Affiliations:** Institute of Immunology, Faculty of Medicine, RWTH Aachen University Hospital, Pauwelsstraße 30, 52074 Aachen, Germany; hannah.trojan@rwth-aachen.de (H.E.T.); jana.jakobs@rwth-aachen.de (J.J.)

**Keywords:** lead, zinc, IL-2, CREM, T cells

## Abstract

Lead, a prevalent heavy metal, impairs the immune system by affecting T cell function. Similarly, zinc deficiency adversely affects T cells, with zinc deficiency and lead exposure being linked to reduced interleukin-2 (IL-2) production. Zinc deficiency has been associated with increased expression of the transcription factor CREM 100 kDa, which downregulates IL-2. Previous research suggests zinc may mitigate lead’s toxic effects. This study explored the molecular mechanism underlying IL-2 reduction in lead-exposed T cells and examined the role of zinc status. The effects of lead exposure were investigated in Jurkat T cells in zinc-adequate, zinc-deficient, and zinc-supplemented conditions. Results showed that lead exposure increased CREM 100 kDa expression, which was amplified under zinc-deficient conditions. Consequently, IL-2 production was significantly lower in cells exposed to both lead and zinc deficiency compared to lead exposure alone. However, zinc supplementation counteracted these effects, preventing CREM 100 kDa overexpression and restoring IL-2 levels. In conclusion, we identified CREM 100 kDa as a potential molecular mechanism behind the lead-induced IL-2 decrease in Jurkat T cells, with zinc deficiency exacerbating this effect. These findings highlight the protective role of zinc in counteracting lead toxicity and emphasize the importance of maintaining adequate zinc levels for immune health.

## 1. Introduction

Lead is a widespread heavy metal that poses a significant risk to human health. It is currently ranked in second position in the substance priority list of toxic substances and disease registry [1]. There is no lowest safe concentration [2], and it was the cause of half of the 2 million deaths due to known chemical exposure in 2019 [3]. It can be found in the Earth’s crust, but the environment is contaminated in general. Sources of exposure have changed over time. While lead was commonly used in gasoline and paints, now occupational exposure, for example, in manufacturing, is a frequent reason for exposure in adults. For children, who are especially vulnerable, the living area poses the highest risk [2].

A vast number of health effects have been observed. Lead can affect all organ systems. Among them, neurological, cardiovascular, renal, and immunological effects have been extensively studied [2]. The immunological effects include a higher susceptibility to chronic infection [4,5], but also autoimmune diseases and allergies [6,7]. Effects occur even in concentrations below 10 µg/dL, which highlights the high sensitivity of the immune system.

Lead alters humoral and cell-mediated immunity [8,9]. For example, it has been seen that lead inhibits interleukin-2 (IL-2) production in Jurkat T cells [10]. IL-2 is an important cytokine for lymphocyte proliferation, differentiation, and homeostasis, which is vastly produced by T helper cells [11].

Zinc is an essential trace element that is important for over 300 enzymes and proper immune function [12]. It also influences IL-2 production. In zinc deficiency, IL-2 production is decreased [13], while zinc supplementation in zinc-deficient elderly [14] or zinc-deficient patients [15] improves IL-2 expression.

There have been studies showing that zinc can counteract the toxic effects of lead. Cerklewski et al. [16] and Kataba et al. [17] demonstrated that dietary zinc intake decreased the severity or toxic lead effects in rats. Wani et al. [18] suggest that higher blood zinc levels are associated with less damage due to lead exposure in occupational workers. Zinc deficiency, on the other hand, seems to increase lead absorption and toxicity [19].

A potential molecular target of zinc to influence lead toxicity is the cyclic adenosine monophosphate (cAMP) responsive element modulator (CREM). The CREM is a transcription factor with over 20 isoforms [20], including activators and repressors [21] of the cAMP signaling pathway. It can function as an early response gene within the cAMP signaling pathway and has been studied in different cell systems [22,23,24]. Our group previously demonstrated that zinc deficiency is associated with an increase in CREMα expression, which is mediated by an elevation in protein phosphatase 2A (PP2A) activity. This CREMα increase was correlated with an IL-2 decrease [25]. Short-term zinc supplementation, in contrast, can reduce CREMα expression and increase IL-2 production in the zinc-deficient elderly [14].

The aim of our study is to investigate the molecular mechanism behind the decreased IL-2 production in lead-exposed cells and the potential effect of different zinc conditions. We hypothesize that lead increases CREM expression, which in turn downregulates IL-2 production. Moreover, we presume that the co-occurrence of zinc deficiency and lead exposure will result in a further reduction in IL-2 production due to CREM expression being upregulated. Finally, we postulate that zinc supplementation can act as a countermeasure against lead-induced changes.

## 2. Results

### 2.1. Lead Exposure Decreases IL-2 Production in PBMCs

Cell lines offer many advantages, but it is substantial to investigate whether the effects are transferable to primary cells. To compare, we studied whether we could see the IL-2 decrease in PBMCs (peripheral blood mononuclear cells) and Jurkat T cells before using Jurkat T cells in further experiments.

**Figure 1:**
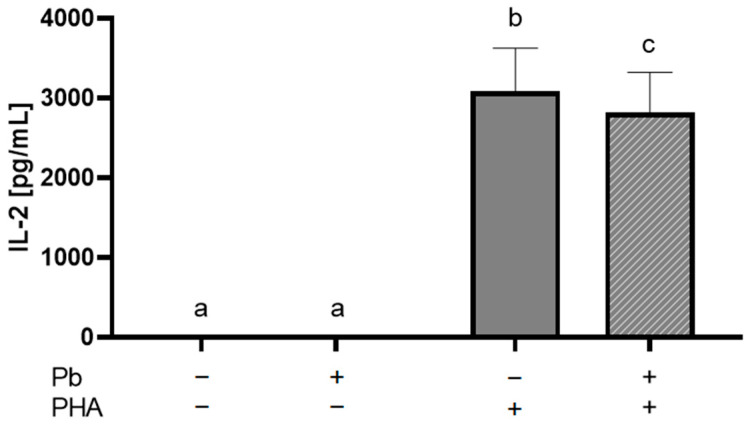
Interleukin-2 production is decreased in lead-exposed PBMCs. PBMCs were incubated without or with 1 µM lead (Pb) for 24 h and then stimulated with 1 µg/mL phytohaemagglutinin (PHA) or left unstimulated. After 24 h, the supernatants were taken, and an interleukin-2 (IL-2) ELISA assay was performed as described in materials and methods. Data are presented as mean + SEM with *n* = 5. Statistical significance was calculated by ordinary one-way ANOVA with the Holm–Sidak post hoc test. When means are not sharing a letter, they are significantly different (*p* < 0.05).

After PMBCs were incubated with lead for 24 h, we stimulated them with 1 µg/mL PHA (phytohemagglutinin) for an additional 24 h. Afterward, we analyzed the supernatants by IL-2 ELISA assay. Neither the unstimulated control nor the lead group produced a measurable amount of IL-2. In PHA-stimulated cells, lead significantly decreased the IL-2 production compared to the lead-unexposed cells (Figure 1). Thus, we conclude that the IL-2 decrease can be reproduced in PBMCs and that Jurkat T cells are a suitable model to study the influence of lead in T cells. Therefore, we conducted further investigation into the Jurkat T cell line.

### 2.2. Optimizing Zinc Conditions and Lead Exposure

Before performing the experiments, we optimized the model to ensure zinc and lead concentrations did not induce measurable cytotoxicity at the levels used in subsequent experiments. To investigate the impact of zinc status on lead-induced IL-2 reduction, Jurkat T cells were cultivated in three different zinc conditions. Different groups should differ significantly in their zinc concentration but not in cell viability.

**Figure 2:**
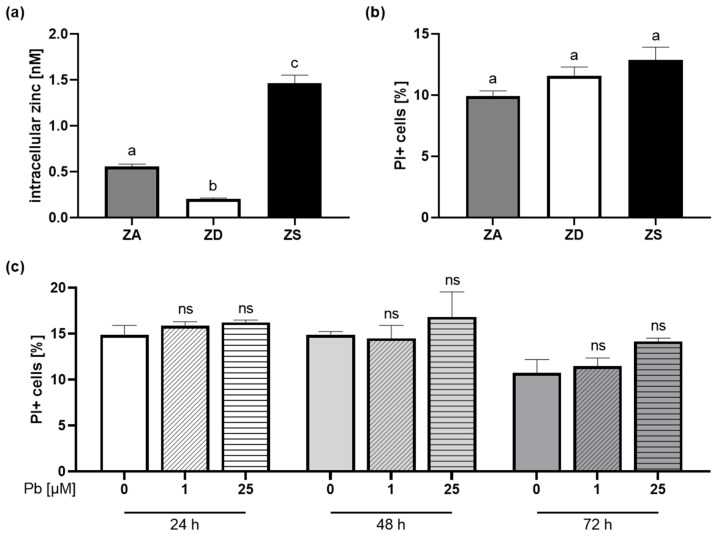
Optimizing zinc conditions and lead exposure. Jurkat T cells were incubated in zinc-adequate (ZA), zinc-deficient (ZD), and zinc-supplemented (ZS) conditions for one week, (**a**) stained with FluoZin-3 AM for intracellular zinc measurements (ZA *n* = 36, ZD *n* = 32, ZS *n* = 6) and (**b**) with propidium iodide (PI) for cell viability (ZA *n* = 39, ZD *n* = 27, ZS *n* = 6). In addition, cells were incubated for 24 h, 48 h, and 72 h without or with 1 µM or 25 µM of lead (Pb) and (**c**) stained with propidium iodide for cell viability to examine the lead-dependent toxicity (*n* = 3). All experiments were performed using flow cytometry. Data are presented as mean + SEM. Statistical significance was calculated by ordinary one-way ANOVA with the Holm–Sidak post hoc test (**a**,**b**) and two-way ANOVA with the Holm–Sidak post hoc test (**c**) with bars not sharing the same letter indicating statistically significant differences (*p* < 0.05, ns: not significant).

Jurkat T cells were incubated in regular Jurkat cell culture medium (zinc-adequate, ZA), Chelex medium (zinc-deficient, ZD), and zinc-supplemented (+30 µM ZnSO_4_) (ZS) conditions for one week, then cell viability with propidium iodide and intracellular zinc concentration with FluoZin-3 AM was measured with flow cytometry. In the zinc-deficient medium, we achieved a significant decrease in intracellular zinc concentration (Figure 2a). Adding 30 µM of ZnSO_4_ increased the zinc concentration significantly compared to the ZA as well as ZD mediums (Figure 2a). The cell viability staining showed no significant differences between all groups (Figure 2b). Hence, the chosen model is suitable because the zinc concentrations significantly differ while the cell viability is not significantly affected.

To scrutinize the effect of different lead concentrations on the survival of Jurkat T cells, we measured the cell viability after 24 h, 48 h, and 72 h incubation in different lead concentrations. For both 1 µM and 25 µM of lead, we saw no significant differences in cell viability across all measured time points (Figure 2c). In all further experiments, we continued with 1 µM of lead.

### 2.3. Lead and Zinc Modulate IL-2 Production in Jurkat T Cells

After the optimal lead and zinc concentrations had been identified, attention was directed toward examining their interactive effects on IL-2 production. We posed the question of whether they influence one another and, more precisely, whether zinc supplementation or deficiency alters the lead-induced IL-2 decrease.

Therefore, Jurkat T cells were incubated for one week in ZA, ZD, or ZS conditions. A total of 1 µM lead was added, and after 24 h, the cells were stimulated with PHA or left unstimulated for another 24 h. We collected the supernatants for IL-2 quantification with an ELISA assay.

**Figure 3:**
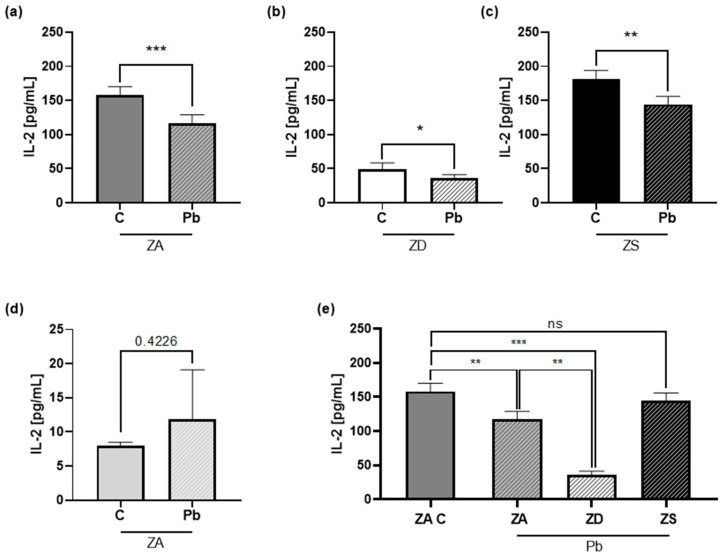
Lead and zinc modulate interleukin-2 (IL-2) production in Jurkat T cells. Jurkat T cells were incubated in (**a**) zinc-adequate (ZA) (*n* = 12–14), (**b**) zinc-deficient (ZD) (*n* = 6), and (**c**) zinc-supplemented (ZS) *(n* = 3) conditions for one week and then without or with 1 µM of lead (Pb) for 24 h. Subsequently, the cells were stimulated with (**a**–**c**) 1 µg/mL phytohaemagglutinin (PHA) or (**d**) left unstimulated (*n* = 3), and after 24 h the supernatants were taken. IL-2 production was measured by ELISA assay. The same data as in (**a**–**c**) are displayed in **e** with an emphasis on the comparison of the different zinc conditions. Data are presented with mean + SEM. Statistical significance was calculated by a two-tailed paired *t*-test (**a**–**d**) and ordinary one-way ANOVA with the Holm–Sidak post hoc test (**e**) (* *p* < 0.05, ** *p* < 0.01, *** *p* < 0.001, ns: not significant).

Lead exposure decreased IL-2 production in every zinc condition compared to the control in PHA-stimulated cells (Figure 3a–c). In unstimulated cells, lead did not significantly affect IL-2 production. However, most of the IL-2 values were below the detection limit of 7.8 pg/mL (Figure 3d). The combination of lead exposure and zinc deficiency decreased IL-2 production even further (Figure 3e). Zinc supplementation could prevent the decrease in IL-2 production compared to the ZA control group (Figure 3e). In summary, lead significantly decreased IL-2 production in every zinc condition. However, the extent of the reduction differed.

### 2.4. Zinc and Lead Affect CREM Expression

Seeing that the zinc conditions modulate the decline of IL-2 production in lead-exposed cells, we wanted to investigate the molecular mechanism for zinc and lead interaction in more depth. A possible molecular target is transcription factors, as there is evidence that heavy metals may have a variety of interactions with transcription factors [26,27]. The transcription factor CREMα has been reported to inversely correlate with zinc concentration, and, therefore, CREMα was investigated in lead-exposed cells in different zinc conditions.

**Figure 4:**
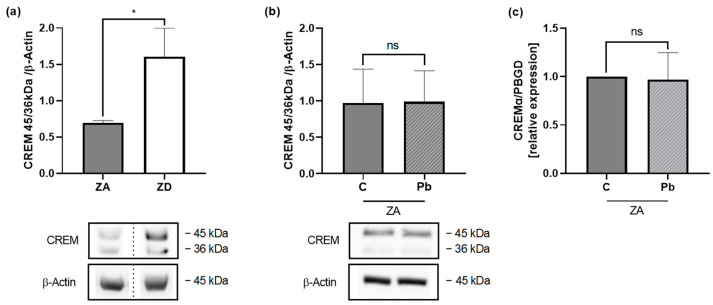
Lead does not increase 45/36 kDa cAMP responsive element modulator (CREM). Jurkat T cells were incubated (**a**) long-term in zinc-adequate (ZA) and zinc-deficient (ZD) medium (*n* = 5). Cells were lysed for Western blot analysis. (**b**) Jurkat T cells were incubated without or with 1 µM of lead (Pb). After 48 h, lysates were collected (*n* = 11) or (**c**) cells were isolated for CREMα RNA analysis by PCR (*n* = 3). In experiments a and b, CREM and β-Actin levels were determined by Western blot analysis. CREM was normalized to β-Actin expression, and representative Western blots are shown. CREM and β-Actin were run on different gels. Western blot (**a**) shows one membrane but not adjacent lanes. Data are presented as mean + SEM. Statistical significance was calculated by a two-tailed paired *t*-test. (* *p* < 0.05, ns: not significant).

We incubated Jurkat T cells in ZA and ZD conditions long-term to confirm the increase in CREM 45/36 kDa in zinc deficiency (Figure 4a). After 48 h of lead exposure in ZA medium, we analyzed the CREM 45/36 kDa bands to look for possible regulation by lead. However, there was neither a regulation of the protein concentration (Figure 4b) nor of CREMα RNA levels (Figure 4c).

While the increase in CREM 45/36 kDa in ZD was reproduced, lead did not affect CREM 45/36 kDA.

To investigate other isoforms, we incubated Jurkat T cells for one week in ZA, ZD, and ZS medium and then exposed the cells to lead for 48 h. Cell lysates were collected for Western blot, and the CREM 100 kDa band was analyzed.

**Figure 5:**
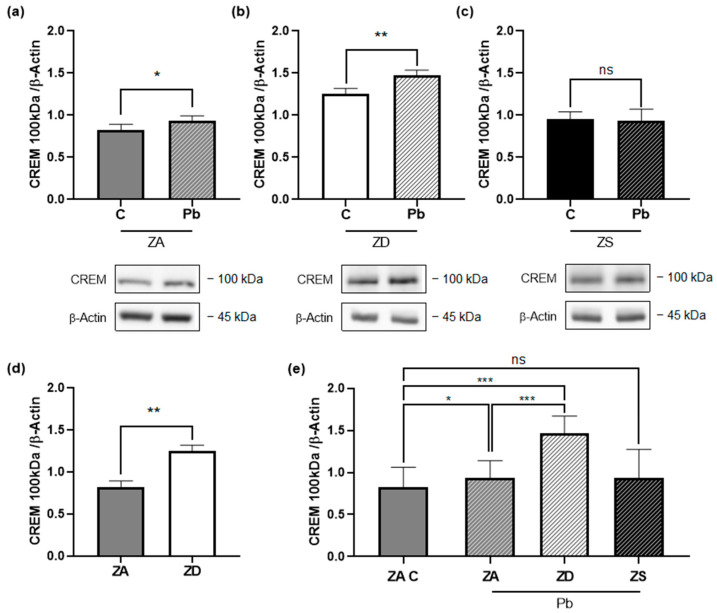
Zinc supplementation prevents cAMP responsive element modulator (CREM) 100 kDa increase in lead-exposed Jurkat T cells. Jurkat T cells were incubated in (**a**) zinc-adequate (ZA) (*n* = 12), (**b**) zinc-deficient (ZD) (*n* = 9), and (**c**) zinc-supplemented (ZS) (*n* = 6) conditions for one week and then without or with 1 µM of lead (Pb). After 48 h, lysates were collected. CREM and β-Actin levels were determined by Western blot analysis. CREM was normalized to β-Actin expression, and representative Western blots are shown. CREM and β-Actin were run on different gels. The same data as in (**a**–**c**) are displayed in (**d**,**e**) to better depict the differences between zinc conditions. Data are presented with mean + SEM. Statistical significance was calculated by a two-tailed paired *t*-test (**a**–**d**) and ordinary one-way ANOVA with the Holm–Sidak post hoc test (**e**) (* *p* < 0.05, ** *p* < 0.01, *** *p* < 0.001, ns: not significant).

We found a significant increase in CREM 100 kDA in lead-exposed ZA cells compared to the control group (Figure 5a). In the ZD group, the increase is even more significant (Figure 5b), whereas there is no significant difference in the ZS group (Figure 5c). ZD itself also increased CREM 100 kDa significantly (Figure 5d).

Equivalent to the IL-2 production analysis, we compared the ZA control group to the lead-exposed cells in the ZA, ZD, and ZS groups to better understand the influence of different zinc conditions on CREM 100 kDA.

As described above, we saw a significant increase in CREM 100 kDa in lead exposed ZA T cells. The combination of lead exposure and zinc deficiency increased CREM 100 kDa even more, but with zinc supplementation, there was no significant difference to the ZA control (Figure 5e). To conclude, lead exposure correlates with higher CREM 100 kDA concentrations in zinc-adequate conditions. While zinc deficiency enhances the increase in CREM 100 kDa, no significant increase was observed in the zinc-supplemented group.

## 3. Discussion

Various negative effects of lead on the immune system are known [2] and thus it is of interest to unravel underlying molecular mechanisms to find potential treatments and preventive measures.

As it is known that zinc is essential for a well-functioning immune system [12], we aimed to investigate the influence of zinc on lead-induced IL-2 decrease in T cells and the molecular mechanism behind it.

**Figure 6:**
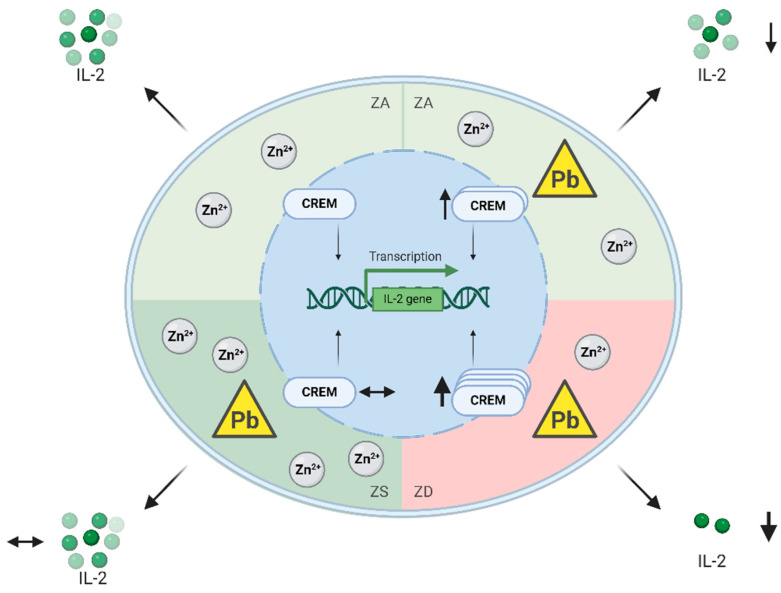
Overview of lead- and zinc-associated changes in IL-2 production and CREM expression. Lead (Pb) increases CREM expression in zinc-adequate (ZA) Jurkat T cells and concurrently lowers IL-2 production. This effect is enhanced by zinc deficiency (ZD) while zinc supplementation (ZS) can prevent the CREM increase and subsequent IL-2 decrease. This figure was created with BioRender.com.

In this study, we observed that Jurkat T cells exposed to lead produced less IL-2, which correlated with an increase in CREM 100 kDa expression. Zinc deficiency enhanced and zinc supplementation reduced the IL-2 decrease and CREM 100 kDa increase (Figure 6). Therefore, we propose that zinc acts as a protective measure against lead-induced downregulation of IL-2 production.

First, we confirmed that the decrease in IL-2 production in lead-exposed Jurkat T cells studied by Colombo et al. could be replicated in PBMCs [10]. The immortalized Jurkat T cell line is derived from a patient with T cell leukemia [28]. Thus, these cells are not completely comparable to in vivo T cells and might express different genes or change cell characteristics. The Jurkat T cell line is commonly used to study T cell biology, including T cell activation, and it has been proven to be useful in understanding this pathway [29]. We were able to validate the lead-induced decrease in IL-2 in PBMC, indicating that the Jurkat T cell line appears to be a suitable model to study changes due to lead exposure.

Next, we optimized the zinc conditions and lead concentration used in the following experiments. Since Colombo et al. used 1 µM of lead [10], and the toxicological profile of lead in the ATSDR reports severe effects on the immune system in blood lead levels (PbBs) of <10 µg/dL (ca. 0.48 µmol/L). We focused on the effect of lower lead concentrations on T cells. It was seen before that zinc deficiency and supplementation can cause apoptosis and decrease the number of living T cells [12]. For lead, the research on T cell abundance is not consistent [2]. Jorissen et al. [30] investigated the impact of Pb(NO_3_)_2_ on PBMCs and could not detect any significant changes in cell viability up to 100 µM lead. Likewise, La Fuente et al. [31] did not see any toxic effects in PBMCs up to 500 µM lead, whereas others saw an inhibition in proliferation [32]. The activation state of the cells might also play an important role, as Colombo et al. [10] observed that lead only increased apoptosis in activated Jurkat T cells. Although a reduced cell number might be part of the immunosuppressive effect of lead, our goal was to design an experimental setup in which zinc and lead did not affect cell viability. Otherwise, changes in IL-2 or CREM concentration could be attributed to the number of living cells.

In humans, the commonly used biomarker for lead exposure is the blood lead level (PbB). As described above, many immunological health effects have been observed in a PbB below 10 µg/dL, which is equivalent to 0.48 µM [2].

Data on dose-related changes in immune function are inconsistent. Most epidemiological studies detect PbB around 50 µg/dL or lower in the lead-exposed groups [33]. This is equal to a low single-digit µM range. Thus, we decided to use 1 µM lead for further experiments.

We used PHA to stimulate the Jurkat T cells. PHA is a lectin that acts as a mitogen and activates T cells. It binds to the TCR/CD3 complex and causes maximum T cell proliferation [34]. Colombo et al. suggested that the T cell activation mode as well as the activation state are necessary to take into consideration when it comes to heavy metal toxicity. Lead seems to perturb the T cell activation downstream of the protein kinase C (PKC) and calcium mobilization when cells are stimulated at the same time or after lead exposure. Stimulation before exposure causes changes upstream of PKC/Calcium [8,10]. We decided to investigate T cell activation after lead exposure to mimic what happens when the immune system of lead-exposed people is activated.

IL-2 is an important cytokine for T helper (Th) cell polarization and proliferation [11]. After stimulation, we saw a decrease in IL-2 production in lead-exposed compared to non-exposed Jurkat T cells. This was also described by Colombo et al. [10]. Iavicoli et al. [9] saw a dose-dependent change in mice fed with different amounts of lead. While the IL-2 concentration decreased in higher doses, there was even an increase in IL-2 in lead concentrations below background values. The findings in humans are also inconsistent. Some research groups saw no change in IL-2 production in lead-exposed workers (above 10 µg/mL PbB) [35], while others observed a lower IL-2 concentration [36]. The differences might be due to differences in the study population, co-exposure to other heavy metals, and duration and dose of lead exposure.

Zinc deficiency caused a decrease in IL-2 production. This has already been shown in several other studies [37]. In our zinc supplementation model, 30 µM of ZnSO_4_ did not change IL-2 expression. Higher zinc concentrations of 200 µM ZnSO_4_ were seen to inhibit IL-2 production via inhibiting calcineurin activity [38]. This shows that an optimal zinc status is important. Zinc deficiency, as well as zinc supplementation above 50 µM, can negatively influence immune function [12].

Without IL-2, the Th1 differentiation is impaired [39]. Th1 expression was also lower in lead-exposed workers [36], and the polarization shifted towards more Th2 cells [8]. Concomitant to the increase in IL-2, Iavicoli et al. [9] saw more Th1 cells in lower lead levels.

Data suggest that transcription factors are a molecular target of heavy metals [26,27]. Colombo et al. [10] investigated the nuclear factor of activated T cells (NFAT) but could not see a correlation between IL-2 production and NFAT activation. They suggest that other transcription factors might be relevant for understanding the IL-2 decrease in lead-exposed cells. Furthermore, they propose a shift to Th2 might be a possible explanation.

Lead was found to upregulate intracellular cAMP (icAMP) levels [8]. cAMP, in turn, can activate CREM [20]. This suggests that the CREM pathway might be modulated by lead. There is a variety of CREM isoforms known, which are produced by alternative splicing [21].

We could detect an increase in CREM 100 kDa in lead-exposed Jurkat T cells. CREM 100 kDa has been described before by Liu et al. [40]. They saw an increase in CREM 100 kDa in patients treated with proton-pump inhibitors (PPI), which affect intracellular zinc distribution, and proposed an alteration of CREM isoforms [40]. Another CREM isoform that has been broadly investigated in T cells is CREMα. CREMα binds to the IL-2 promotor and recruits histone deacetylase 1 (HDAC1), which causes chromatin condensation [41]. Thus, this isoform is acting as a repressor. CREMα levels are higher in zinc deficiency [14,25] and can be downregulated with zinc supplementation [14]. We could also see an increase in CREMα in zinc deficiency, but we did not see any changes in RNA or protein levels after 48 h of lead exposure. Although CREMα is considered to be an early response gene [22], it might be interesting to study the potential long-term effects of lead, precisely changes in CREM regulation and isoforms. In addition, we investigated protein levels (and CREMα RNA levels) and not protein activity. It has been reported that CREM can be activated by phosphorylation, which recruits a co-activator, or it can be active independent of phosphorylation [42]. Thus, looking at CREM activation might give us further insight into the lead-induced changes of the CREM pathway.

The combination of zinc deficiency and lead exposure further reduced IL-2 and increased CREM 100 kDa. In contrast, zinc supplementation could counteract both effects. Zinc and lead might modulate these pathways individually or interact directly with each other. The interaction between lead and zinc has been extensively studied. They seem to act competitively, as it has been seen that lead can decrease zinc absorption just as zinc decreases lead absorption [16,43]. Furthermore, lead can replace zinc on heme enzymes [44], and, on the other hand, many toxic lead manifestations can be reduced by zinc [45]. Lead has been associated with autoimmune diseases [7,8]. In our study, zinc seems to be able to reduce the lead effects on a molecular level, so it should be evaluated whether zinc can act as a prophylactic measure to reduce lead effects such as autoimmune diseases. This might be especially interesting for workers with occupational lead exposure or people living in lead-contaminated areas.

The function of the isoform CREM 100 kDa has not yet been identified. Therefore, further experiments are necessary to fully understand the molecular mechanism of lead exposure in T cells.

Hopkins and Failla [46] saw that copper deficiency can decrease IL-2 by inhibiting its transcription independent of IL-2 mRNA stability. Following that, it would be of interest to examine whether lead regulates IL-2 through CREM by influencing transcription and/or mRNA stability to gain further insight into the process.

Besides CREM, there are other transcription factors that regulate IL-2 production. Commonly known are NFAT, activator protein 1 (AP-1), and nuclear transcription factor—kappa B (NFκB) [47]. Ramesh et al. [48] studied pheochromocytoma cells and found an activation of nuclear transcription factor—kappa B (NFκB) and activator protein 1 (AP-1) in lead-exposed cells but did not measure IL-2. As previously described, there is conflicting data on whether IL-2 is increased or decreased with lead exposure. Therefore, it might be useful to investigate NFκB and AP-1 in future studies to see if the IL-2 changes can be explained by these transcription factors independently of zinc. Other interesting targets are RhoA and B lymphocyte-induced maturation protein-1 (BLIMP-1). RhoA, a GTPase, inhibited IL-2 in Jurkat T cells, and BLIMP-1 was seen to repress IL-2 in T cells in mice [49,50]. These could also be influenced by lead, which should be further studied to get a better understanding of the lead-induced changes in T cells.

Moreover, it must be taken into consideration that the use of cell culture media is known to influence the effects of metals as its buffering capacity is less in comparison to in vivo conditions. Furthermore, FCS, which plays a significant role in buffering, varies across suppliers. Consequently, the metal speciation cannot be precisely predicted, and the concentrations that demonstrated effects in the experiments may differ substantially from those under physiological conditions [51]. Considering this and the fact that cell lines are not necessarily optimal surrogates for all aspects of the human T cell function, future studies may investigate the zinc and lead interactions in PBMCs and later in lead-exposed humans.

Our in vitro data suggest that zinc can counteract the lead-induced IL-2 decrease. Thus, it would be interesting to investigate whether zinc supplementation can improve immune function and lower the susceptibility to infections in lead-exposed patients.

In conclusion, we identified CREM 100 kDa as a molecular target that is increased in lead-exposed Jurkat T cells and could be responsible for the decreased IL-2 expression. We show that zinc deficiency amplifies and zinc supplementation can prevent the lead-induced IL-2 decrease and CREM 100 kDa increase. This underlines the role of zinc in diminishing lead toxicity and emphasizes zinc as a protective measure against lead-induced disruption of normal immune function.

## 4. Materials and Methods

### 4.1. Study Design

First, we investigated if the results of Colombo et al. were transferable to peripheral blood mononuclear cells (PBMCs) [10]. For that reason, PBMCs were isolated and incubated for 15 min at 37 °C and 5% CO_2_. Then, 1 µM of lead (Pb(NO_3_)_2_ was added, or the cells were left untreated. After incubation for 24 h, the cells were stimulated with 1 µg/mL phytohaemagglutinin (PHA, Genaxxon bioscience GmbH, Ulm, Germany) or left unstimulated. Following an additional 24 h of incubation, the supernatants were collected for IL-2 ELISA.

For the rest of the experiments, we used Jurkat T cells. They were incubated in zinc-adequate (ZA), zinc-deficient (ZD), and zinc-supplemented (ZS) medium for one week (if not otherwise stated) and then adjusted to a concentration of 1 ×10^6^ cells/mL. A total of 1 µM lead was added or Jurkat cells were left untreated. Equivalent to the PBMC stimulation, Jurkat cells were incubated for 24 h and stimulated with 1 µg/mL PHA or left unstimulated. After an additional 24 h of incubation, supernatants and cell lysates (preparation described under “Western blot” or “PCR”) were collected for IL-2 ELISA and CREM Western blot or CREMα PCR as described. ELISA and Western blot samples were stored at −20 °C, and PCR samples at −80 °C.

### 4.2. PBMC Isolation

We collected venous blood from healthy young volunteers with ethics committee approval (RWTH Aachen University Hospital, document No. EK 23-234) and added 50 U/mL sodium heparin (B. Braun, Melsungen, Germany). The blood was diluted 1:2 with phosphate-buffered saline (PBS) (Sigma-Aldrich, St. Louis, MO, USA), and we performed a density centrifugation over Lymphocytes Separation Media with a density of 1.077 g/mL (Capricorn Scientific, Ebsdorfergrund, Germany). The PBMCs were collected from the interphase, washed three times, and then adjusted to a concentration of 1 × 10^6^ /mL in RPMI 1640 medium (Sigma-Aldrich, St. Louis, MO, USA) containing 10% heat-inactivated fetal calf serum (FCS) (Bio&Sell, Feucht, Germany), 2 mM L-glutamine, 100 U/mL potassium penicillin, and 100 µg/mL streptomycin sulfate (all Sigma-Aldrich, St. Louis, MO, USA).

### 4.3. Cell Culture and Zinc Deficiency/Substitution Models

The human T lymphocyte cell line Jurkat was cultivated at 37 °C in 5% CO_2_ in a cell culture medium containing RPMI 1640 medium (Sigma-Aldrich, St. Louis, MO, USA) supplemented with 10% heat-inactivated FCS, 2 mM L-glutamine, 100 U/mL potassium penicillin,100 µg/mL streptomycin sulfate, 1 mM sodium pyruvate, and 1% 100x nonessential amino acids (all Sigma-Aldrich, St. Louis, MO, USA).

To obtain a zinc deficiency model, Chelex 100 Resin (Sigma-Aldrich, St. Louis, MO, USA) was used. As Mayer et al. [52] described, Chelex 100 Resin contains paired iminodiacetate ions, which chelate metal ions, especially divalent ions like zinc. The medium was treated with Chelex 100 Resin for one hour. Afterward, 500 µM CaCl_2_ (Merck, Darmstadt, Germany) and 400 µM MgCl_2_ (Sigma-Adrich, St. Louis, MO, USA) were readded, and the pH was adjusted to 7.4. The zinc-deficient medium was filter-sterilized, and the zinc concentration was measured by atomic absorption spectrometry (AAnalyst 800, PerkinElmer, Waltham, MA, USA).

For the zinc-supplemented model, 30 µM of ZnSO_4_ (Sigma-Adrich, St. Louis, MO, USA) was added to the normal cell culture medium.

### 4.4. Toxicity Test

We measured the cell viability via propidium iodide (PI) staining. Therefore, 5 × 10^5^ Jurkat T cells were diluted in 500 µL PBS and stained with 10 µg/mL PI for 10 min at 4 °C in the dark. Afterward, the cells were washed and then resuspended in 300 µL PBS. Flow cytometry measurements were performed using a FACSCalibur (BD, Franklin Lakes, NJ, USA). All samples were analyzed with FlowJo v10 (BD, Franklin Lakes, NJ, USA) and gated equally.

For the toxicity testing, Jurkat T cells were adjusted to a concentration of 5 × 10^4^ cells/mL and seeded in 24-well plates in a cell culture medium. We added 0, 1, and 25 µM of Pb(NO_3_)_2_ (Sigma-Aldrich, St. Louis, MO, USA) and incubated the cells at 37 °C and 5% CO_2_ for 24 h, 48 h, and 72 h. Cell viability was checked as described above. Cell cultures with over 20% PI-positive cells were excluded from further experiments.

### 4.5. Flow Cytometric Measurement of Intracellular Free/Labile Zinc

According to Haase et al. [53], 1 × 10^6^ cells were incubated in 500 µL PBS and 1 µM FluoZin-3 AM (Invitrogen, Waltham, MA, USA) for 30 min in a 37 °C water bath in the dark. The cells were washed with PBS twice and resuspended in 900 µL PBS. Each sample was divided into 3 aliquots containing either (1) the untreated sample (F), (2) the sample plus 50 µM the zinc chelator N,N,N′,N′-tetrakis(2-pyridinylmethyl)-1,2-ethanediamine (TPEN, Sigma-Adrich, St. Louis, MO, USA) (minimal fluorescence, Fmin), or (3) the sample plus 100 µM ZnSO_4_ and 25 µM pyrithione (Sigma-Adrich, St. Louis, MO, USA) (for maximal fluorescence, Fmax). After another incubation for 10 min in a 37 °C water bath in the dark, flow cytometry measurements were performed using FACSCalibur. All samples were gated equally, and only structurally normal (intact) cells were included. The intracellular zinc concentration was calculated using [Zn^2+^]= K_D_ × [(F − Fmin)/(Fmax − F)] with the dissociation constant K_D_ of 8.9 nM for the FluoZin-3 AM/Zn^2+^ complex [54].

### 4.6. IL-2 Quantification

IL-2 protein concentration in supernatants of PBMCs and Jurkat T cells was determined by OptEIA^TM^ ELISA assay (BD, Franklin Lakes, NJ, USA) according to the manufacturer’s instructions. The detection limit was 7.8 pg/mL. If the concentration values were below this value, the values were set just below the detection limit. For PBMCs, the stimulated samples were diluted. The absorption was measured using a Spark microplate reader (Tecan, Männedorf, Switzerland).

### 4.7. Western Blotting

A total of 1 × 10^6^ Jurkat T cells were centrifuged and resuspended in sampling buffer (65 mM Tris-HCl (pH 6.8) (Roth, Karlsruhe, Germany), 2% [*w*/*v*] SDS (Merck, Darmstadt, Germany), 26% [*v*/*v*] glycerol Roth, Karlsruhe, Germany) with 1 µM sodium orthovanadate (Sigma-Adrich, St. Louis, MO, USA), 1% β-mercaptoethanol (Merck, Darmstadt, Germany), and 2% protease inhibitor cocktail (Boster Biological Technology, Pleasanton, CA, USA). Cells were lysed with a Vibra Cell sonicator (Sonics & Materials, Newtown, PA, USA) and heated at 95 °C for 3 min. The cell lysates were stored at −20 °C until further use. Before Western blotting, 2% bromophenol blue (Merck, Darmstadt, Germany) was added, and the cells were heated at 95 °C for 3 min again. A 10% polyacrylamide gel was used for protein separation. A total of 3 µL of colored prestained standard (New England BioLabs, Frankfurt am Main, Germany) for molecular weight determination and 10 µL of samples were added per lane and separated at 170 V. Then, samples were blotted on nitrocellulose membranes with a pore size of 0.45 µM at 100 V for 75 min. Ponceau S (PanReac, AppliChem, Darmstadt, Germany) was used for staining to check loading before the membranes were washed in TBS-T (20 mM Tris (pH 7.6) 137 mM NaCl, and 0.1% [*v*/*v*] Tween 20) for 5 min. Subsequently, the membranes were blocked for 1 h in TBS-T plus 5% fat-free dry milk and washed in TBS-T 3 times. β-Actin (Cell Signaling Technology, Danvers, MA, USA) (diluted 1:1000 in TBS-T + 5% BSA) and CREM C-2 (Santa Cruz Biotechnology, Dallas, TX, USA) (diluted 1:500 in TBS-T + 5% BSA) were utilized as primary antibodies. The membranes were incubated in those primary antibodies at 4 °C overnight. On the second day, the membranes were washed 3 times with TBS-T and incubated for 3 h in the secondary antibodies at room temperature. Anti-mouse HRP-linked antibody for CREM C-2 and anti-rabbit HRP-linked antibody for β-actin (both Cell Signaling Technology, Danvers, MA, USA) were both diluted 1:2000 in TBS-T + 5% fat-free dry milk. Lastly, the membranes were washed 3 times and detected with Westar Antares (Cyanagen, Bologna, Italy) using LAS-3000 (Fujifilm Lifescience, Tokyo, Japan).

We analyzed the band density with ImageJ (Version 1.53t, NIH, Bethesda, MD, USA).

### 4.8. Quantitative Polymerase Chain Reaction (qPCR)

A total of 1 × 10^6^ Jurkat T cells were centrifuged at 300× *g* for 5 min and resuspended in 1 mL EXTRAzol reagent (blirt, Danzig, Poland). After 5 min of incubation at room temperature, the samples were stored at −80 °C. mRNA was isolated according to the manufacturer’s instructions with the following modifications: After the addition of chloroform, the samples were incubated for 10 min; after the addition of 75% ethanol, the samples were centrifuged at 12,000× *g* for 5 min; and after resuspension in Diethyl pyrocarbonate (DEPC)-treated water, they were incubated at 60 °C for 10 min. The RNA concentrations of the samples were determined by using Nanodrop One (Thermo Fisher Scientific, Waltham, MA, USA). cDNA synthesis was performed using a qScript cDNA synthesis kit (Quantabio, Beverly, MA, USA) following the manufacturer’s instructions. Fluorescent Power SYBR^TM^ green PCR Master Mix and Quant studio 3 system (both Applied biosystems by Thermo Fisher scientific, Waltham, MA, USA) were used for qPCR. The following primers were used: CREMα (forward primer: TCG CGA ACT TGG GAC GA, reverse primer: CTG ACC TAA AGCA GGG ATTG), and porphobilinogen deaminase (PBGD) (forward primer: ACG ATC CCG AGA CTC TGC TTC, reverse primer: GCA CGG CTA CTG GCA CAC T). Initial denaturation was at 95 °C for 10 min, followed by an annealing temperature of 95 °C for 15 s and 60 °C for 1 min for 40 cycles. Subsequently, a melting curve was performed. Aqua ad injectabilia (Braun, Melsungen, Germany) was used as a no-template control, and all samples were analyzed in duplicates.

The ∆∆C_T_ method was employed to quantify CREMα expression normalized to PBGD expression [55].

### 4.9. Statistics

GraphPad Prism (version 8.0.1) was used for calculating statistical significance. Experiments were performed at least three times. Outliers were determined and removed accordingly. Normality testing was performed with the Shapiro–Wilk test. The different tests for statistical testing are named in the figure legends. *p* < 0.05 was considered significant (*, ** *p* < 0.01, *** *p* < 0.001). When the mean of each column was compared with the mean of every other column, significances were indicated with letters. Mean values not sharing any letter are significantly different. All graphs were created with GraphPad Prism (version 8.0.1).

## Data Availability

The datasets generated during and/or analyzed during the current study are available from the corresponding author upon reasonable request.
